# PANCREATIC NEUROENDOCRINE TUMORS: SURGICAL RESECTION

**DOI:** 10.1590/0102-672020180001e1428

**Published:** 2019-02-07

**Authors:** Marcos BELOTTO, Bruna do Nascimento Santos CROUZILLARD, Karla de Oliveira ARAUJO, Renata D’Alpino PEIXOTO

**Affiliations:** 1 Depa, German Hospital Oswaldo Cruz;; 2 Faculty of Medical Sciences, Santa Casa de São Paulo;; 3 Oncology Service, Nove de Julho University, São Paulo, SP, Brazil

**Keywords:** Neuroendocrine tumor, Pancreatic neoplasm, Pancreas, Tumores neuroendócrino, Neoplasias pancreática, Pâncrea

## Abstract

**Introduction:**

Pancreatic neuroendocrine tumors (pNET) correspond to about 3% of all tumors in pancreas and could be presented as a difficult diagnosis and management.

**Objective:**

To review the diagnosis and treatment of the pNET available in scientific literature.

**Method:**

A bibliographic survey was performed by means of an online survey of MeSH terms in the Pubmed database. A total of 104 articles were published in the last 15 years, of which 23 were selected as the basis for the writing of this article.

**Results:**

pNET is an infrequent neoplasia and their incidence, in USA, is about 1:100.000 inhabitants/year. Thereabout 30% of them produce hormones presenting as a symptomatic disease and others 70% of the cases could be silent disease. Magnetic Resonance Imaging (MRI) and/or Computed Tomography (CT) have similar sensitivy to detect pNET. They are very important when associated to nuclear medicine mainly Positron Emission Tomography (PET-CT) Gallium-68 to find primary tumor and its staging. The appropriate treatment should be chosen based on characteristics of the tumor, its staging and associated comorbidities.

**Conclusion:**

The surgical resection is still the best treatment for patients with ressectable pancreatic NETs. However, the size, grade, tumor functionality, stage and association with multiple endocrine neoplasia type 1 (MEN-1) are important to define who will be eligible for surgical treatment. In general, tumors bigger than 2 cm are eligible for surgical treatment, except insulinomas whose surgical resection is recommended no matter the size.

## RESUMO

**Introdução:**

Os tumores neuroendócrinos pancreáticos (TNE-P) correspondem a cerca de 3% de todas as neoplasias no pâncreas e são de diagnóstico e manejo difíceis.

**Objetivo:**

Revisar o diagnóstico e tratamento desses tumores disponíveis na literatura científica atual.

**Método:**

Foi realizado levantamento bibliográfico por meio de pesquisa online de MeSH terms na base de dados do Pubmed. Ao todo foram analisados 104 artigos publicados nos últimos 15 anos, dos quais 23 foram selecionados como base para a redação deste artigo.

**Resultados:**

TNE-P é neoplasia pouco frequente com incidência estimada em 1: 100.000 habitantes/ano. Cerca de 30% produzem hormônios, ditos funcionantes, que se apresentam como doença sintomática e outros 70% podem se apresentar como doença silenciosa. Como recurso diagnóstico o uso de tomografia computadorizada ou ressonância nuclear magnética apresentam níveis de sensibilidade parecidas. Sua associação à recursos da medicina nuclear são de grande valia para localização de tumores primários e estadiamento, destacando-se o uso da tomografia por emissão de pósitrons (PET-CT) com Gálio-68. A decisão terapêutica deve ser baseada nas características tumorais, estadiamento e comorbidades associadas.

**Conclusão:**

A abordagem cirúrgica permanece como a terapia de escolha para pacientes com TNE-P ressecáveis. No entanto, a seleção de pacientes para tratamento cirúrgico deve seguir critérios baseados em funcionalidade do tumor, grau, estágio e associação com neoplasia múltipla tipo 1. De forma geral, tumores maiores de 2 cm têm indicação cirúrgica, excetuando-se os insulinomas, cuja proposta cirúrgica independe de seu tamanho.

## INTRODUCTION

First described in 1907 by Siegfried Oberndorfer, neuroendocrine tumors (TNE, or carcinoids, as first denominated) can originate from several sites, predominating in the gastrointestinal tract (60%) and lungs (25%)^23^. Gastroenteropancreatic neuroendocrine tumors are challenging to manage because they represent a heterogeneous family of neoplasms. Although the growth and progression of most of these tumors are slow, it is not uncommon for metastases to occur, which decreases overall survival^23^. Clinical evaluation of gastroenteropancreatic neuroendocrine tumors is dependent on parameters such as degree of differentiation and primary site^3^ ^,^ ^22^. Well-differentiated tumors tend to be more indolent and have a lower chance of metastasis, while the poorly differentiated tumors have a high tendency to develop metastases and behave more aggressively^16^. The location of the primary tumor is also an important tool for assessing the clinical course of the disease. For example, neuroendocrine tumors of the small intestine tend to behave more indolently, whereas the colon and pancreatic primates present less survival^23^. Since pancreatic neuroendocrine tumors (pNET) have distinct molecular, functional, prognostic and treatment characteristics when compared to non-pancreatic NETs, they are usually analyzed separately. pNET, originating from endocrine pancreatic tissues, has an incidence of less than 1: 100,000 inhabitants per year in the United States, which corresponds to about 3% of all pancreatic tumors^7^. About 30% of the pNET are classified as functioning, since they are accompanied by hormonal symptoms, more commonly resulting from the production of insulin or gastrin^8^. Other relevant types are VIPomas, glucagonomas and somatostatinomas. The clinical manifestation depends on the type of hormone or neuropeptide produced by neoplasia. However, 70% of the cases correspond to non-functioning tumors, which also produce a series of substances, but without corresponding to any hormonal syndrome^8^ ^,^ ^17^. The clinical characteristics of the major functioning TNE-P are shown in Table 1. Most of them are sporadic, while some may be associated with hereditary endocrinopathies, including MEN-1, von Hippel Lindau syndrome, neurofibromatosis type 1 and tuberous sclerosis. More frequently gastrinomas and insulinomas may be associated with MEN-1^8^ ^,^ ^17^. 

**TABLE 1:** Clinical presentation of functioning NETs (Jensen et al, 2012^3^ and Sara Massironi et al, 2008^16^)

Tumor	hormone	Localization	Malignancy (%)	Metastasis (%)	Clinical presentation	men-1^#^ (%)
Insulinoma	Insulin	Pancreas (>99%)	<10	10	Episodic Hypoglycemia	8-10
Gastrinoma	Gastrin	Duodenum (70%) Pancreas (25%)	60-90	60	Peptic ulcers, diarrhea, GERD*	30
VIPoma	Vasoactive intestinal peptide (VIP)	Pancreas (90%	40-70	70	Severe diarrhea, hypokalemia, hypochlorhydrya	Rare
Glucagonoma	Glucagon	Pancreas (100%)	50-80	60	Cutaneous rash, diabetes, weight loss, anemia	Rare
Somatostatinoma	Somatostatin	Pancreas (55%) Midgut (44%)	>70	84	Diarrhea, diabetes, gallstone disease, weight loss	Unassociated

^#^ Multiple Endocrine Neoplasia Type 1; *GERD: Gastroesophageal reflux disease

In addition to the nominal classification from the syndromes resulting from the presence of the P-NETs, ​​the World Health Organization classifies them into two general categories regarding aggressiveness: well-differentiated and poorly differentiated^10^. The former have a fairly uniform, solid, trabecular, spiral or glandular pattern nucleus, in addition to pepper-salt chromatin and granular cytoplasm. They are divided into two categories, according to the proliferation rate: low grade (grade 1) and intermediate grade (grade 2) (Table 2). Seconds are all high grade (grade 3), presenting highly aggressive behavior and clinical manifestation similar to neuroendocrine carcinomas of small or large lung cells (Table 2). Some patients have histologically well or moderately differentiated tumors, but with a Ki-67> 20% index, classified in the high grade category. The clinical behavior of these tumors appears to be between mildly differentiated neuroendocrine carcinoma and intermediate-grade tumors^18^.

**TABLE 2:** The classification of NET by the World Health Organization^7^

	Tumor well differentiated		Carcinoma well differentiated		Carcinoma poorly diferentiated
	BB^α^	MB^β^	Grade 1	Grade 2	Grade 3
Size	< 2 cm	> 2 cm	-	-	-
Mitoses number^€^	< 2	> 2	< 2	2 a 20	> 20
Ki-67 index	< 2%	> 2%	< 3%	3 a 20%	> 20%
Vascular invasion	no	Yes	Vascular invasion + metastasis		Perineural/vascular invasion

BB^α^=Benign behavior; MB^β^=malignant behavior. ^€^Mitoses expressed as number / 10 field of high powe

## METHOD

A literature review of the last 15 years of the medical literature was conducted through an online survey of MeSH terms “neuroendocrine tumors” AND “pancreas” AND “treatment”. We included original articles and review articles that addressed the diagnosis and especially the treatment of pancreatic neuroendocrine tumors. We excluded articles that consisted of studies whose object was not similar to the one described above, articles that only addressed clinical and non-surgical treatments. Other articles were used for contextualization and discussion. A total of 104 articles were analyzed, of which 23 were used for the construction of this article

### Image and laboratory 

To define the treatment strategy, imaging and histopathological analysis should be performed. To obtain a histological examination, a biopsy can be performed by fine or gross needle aspiration and analysis of the collected tumor material. This feature can confirm the presence of neuroendocrine tumor, identify regional lymphadenopathy, and help determine proliferation index (number of mitoses, Ki-67), which will be useful in lesion staging and therapeutic decision making.

For analysis of tumor size and study of possible invasion of adjacent structures, magnetic resonance imaging (MRI) and/or computed tomography (CT) are indicated. CT is less sensitive for the detection of primary pancreatic tumors when compared to MRI, but has a higher specificity^20^. For the evaluation of liver metastases, MRI is preferentially indicated, since isolated CT may fail 20% of the time in the detection of hepatic metastatic tumors when compared to MR^1^.

The combination of conventional radiology features and nuclear medicine imaging is often mandatory for visualization of the primary tumor, its staging, and for defining the therapeutic strategy. In Brazil, all modalities of nuclear medicine imaging are not yet available, the most important being somatostatin receptor scintigraphy (SRS, also known as OctreoScan), PET-CT Gallium-68 and PET-CT FDG.

SRS has sensitivity of 80% for well differentiated tumors (grades 1 and 2); however, it presents lower sensitivity for tumors with a diameter smaller than 1 cm, non-metastatic insulinomas^21^ and TNEs with high Ki-67^1^ index. Positron emission tomography/computed tomography (PET-CT) with Gallium-68 has a greater sensitivity for the detection of NSC-P than SRS, reaching more than 90%^21^. PET-CT with fluorodeoxyglucose has better uptake in more aggressive TNEs and less efficacy in tumors grade 1 and 2 due to its limited growth rate^21^. Imaging evaluation is recommended for patients with metastatic disease (more common in the liver and less in the retroperitoneal lymph nodes and bones)^19^, as well as those with no metastatic disease.

Some patients may present with hormone syndromes suggestive of NET, but no tumoral evidence for conventional imaging tests (CT or MRI). For these patients, endoscopic ultrasonography or arterial stimulation and venous sampling may be used. The use of conventional imaging features and endoscopic studies for non-functioning hidden pNET increased preoperative detection to levels close to 100%^9^.

Laboratorially, for non-functioning tumors, the analysis of chromogranin A (CgA) is the most performed, since it is secreted by all types of gastroenteropancreatic neuroendocrine tumors. Their levels are increased in about 70% of the cases of functioning and non-functioning pNET^14^. Attention must be paid to factors that may influence false positive CgA results, such as the use of proton pump inhibitor drugs^11^. Another non-specific marker for non-functioning pNET is the pancreatic polypeptide, which when compared to the use of CgA alone increases the diagnostic sensitivity from 63% to 93%^14^. For functioning pNET, CgA levels will be elevated in approximately 75% of patients and the hormone secreted by the tumor (insulin, glucagon, etc.) functions as a specific tumor marker^11^.

### Treatment

The surgical option is the only potentially curative treatment for pNET, both for functioning and non-functioning tumors. The selection of patients for surgical treatment should follow criteria based on tumor functionality, degree, stage and association with MEN-1.

Patients with gastrinoma and non-MEN-1 carriers should be surgically treated, with or without tumor imaging. In a recent study, survival was >98% for those who underwent surgical resection^13^. In those with gastrinoma and carriers of MEN-1, the approach is controversial. For tumors <2 cm, surgical treatment is not indicated, since patients present almost 100% survival rate in 15 years^13^. In addition, duodenopancreatectomy is a procedure with considerable potential for postoperative complications. For tumors >2 cm tumor enucleation is recommended and duodenopancreatectomy is reserved only for selected cases^6^ ^,^ ^8^.

The National Comprehensive Cancer Network reserves the widest surgical guideline for gastrinoma as well as the clinical management of gastric hypersecretion with the use of proton pump inhibitor drugs and the use of somatostatin analogues (octreotide or lanreotide) as the first choice. For occult tumors, there are two types of conduct: observation and surgical exploration. The surgical option should include duodenotomy and use of intraoperative ultrasound. If the tumor is identified, its enucleation associated with lymph node resection is recommended. In these cases, the surgical procedure is done according to the location of the tumor. When located in the head of the pancreas, the degree of tumor invasion and its proximity to the main pancreatic duct should be studied. When the tumor is judged to be non-invasive, enucleation associated with lymph node resection should be performed. When it is a deep or invasive tumor, near the main pancreatic duct, the recommended course is duodenopancreatectomy. When the tumor is located in the pancreatic body or tail, we recommend distal pancreatectomy with or without splenectomy^12^.

For insulinomas, in patients with or without MEN-1, the indication is surgical. Cure is obtained in 98-100% of cases^2^. The conduct of choice depends on the degree of tumor invasion and the proximity of the main pancreatic duct. For exophytic or peripheral tumors located in the head or pancreatic body, videolaparoscopic enucleation may be considered. In the deep, invasive and near the main pancreatic duct, more aggressive conduct is recommended; when located in the head of the pancreas, duodenopancreatectomy is indicated, while in the distal, distal pancreatectomy is recommended. The videolaparoscopic technique can be considered in these cases^12^.

For non-functioning pancreatic tumors, a surgical procedure is usually recommended^7^. Exception occurs in patients with MEN-1 and who have tumors <2 cm, when the operation is not consensually recommended. For some authors and societies, observation and follow-up should be performed when the tumor is <1 cm, asymptomatic and incidental^12^. The decision for surgical treatment should be made based on the estimated surgical risk, tumor location and comorbidities. The surgical choice may vary between enucleation, distal pancreatectomy and duodenopancreatectomy always associated with regional nodal resection due to the real chances of lymph node metastasis, even in tumors with a size between 1-2 cm^12^.

For patients with tumors >2 cm, the choice is tumor cell excision. The location is indicative for the choice of technique, which may range from duodenopancreatectomy to the distal pancreatectomy associated with splenectomy. Both should be accompanied by lymph node resection due to the risk of metastases^5^ ^,^ ^12^. Complete resection R0 should always be the primary endpoint. Regardless of tumor size, there is no evidence to support adjuvant systemic therapy for pNET and optimal follow-up of patients undergoing surgical treatment remains unknown.

## CONCLUSION

The surgical approach remains the therapy of choice for patients with resectable pNET. However, selection for surgical treatment should follow criteria based on tumor functionality, grade, stage and association with NEM-1. Gastrinomas >2 cm has surgical resection as the treatment of choice, being associated or not with NEM-1. Due to the high cure rates obtained with the surgical intervention, the insulinomas have resection the treatment of choice. For non-functioning tumors, when their size exceeds 2 cm, surgical treatment is recommended, with the patient having or not having type 1 multiple endocrine disease. 

## References

[1] Binderup T, Knigge U, Loft A, Mortensen J, Pfeifer A, Federspiel B, Hansen CP, Hojgaard L, Kjaer A (2010). Functional imaging of neuroendocrine tumors: a head-to-head comparison of somatostatin receptor scintigraphy, 123I-MIBG scintigraphy, and 18F-FDG PET. Journal of Nuclear Medicine.

[2] Crippa S, Partelli S, Zamboni G (2014). Incidental diagnosis as prognostic factor in different tumor stages of nonfunctioning pancreatic endocrine tumors. Surgery.

[3] Dias AR, Azevedo BC, Alban LBV, Yagi OK, Ramos MFKP, Jacob CE, Barchi LC, Cecconello I, Ribeiro U, Zilberstein B (2017). Gastric neuroendocrine tumor: review and update. Arq Bras Cir Dig.

[4] Falconi M, Bartsch DK, Eriksson B (2012). ENETS Consensus Guidelines for the Management of Patients with Digestive Neuroendocrine Neoplasms of the Digestive System: Well-Differentiated Pancreatic Non-Functioning Tumors. Neuroendocrinology.

[5] Ma Falconi, Bb Eriksson, Gc Kaltsas, Bartsch DKd, Je Capdevila, Mf Caplin, Bg Kos-Kudla, Dh Kwekkeboom, Gi Rindi, Gj Klöppel, Nk Reed, Rl Kianmanesh, Jensen RTm, Vienna Consensus Conference participantsn (2016). Consensus guidelines update for the management of functional p-NETs (F-p-NETs) and non-functional p-NETs (NF-p-NETs). Neuroendocrinology.

[6] Giesel FL, Kratochwil C, Mehndiratta A, Wulfert S, Moltz JH, Zechmann CM, Kauczor HU, Haberkorn U, Ley S (2012). Comparison of neuroendocrine tumor detection and characterization using DOTATOC-PET in correlation with contrast enhanced CT and delayed contrast enhanced MRI. Eur J Radiol.

[7] Halfdanarson TR, Rabe KG, Rubin J (2008). Pancreatic neuroendocrine tumors (PNETs) Incidence, prognosis and recent trend toward improved survival. Ann Oncol.

[8] Jensen RT, Cadiot G, Brandi ML (2012). ENETS Consensus Guidelines for the management of patients with digestive neuroendocrine neoplasms functional pancreatic endocrine tumor syndromes. Neuroendocrinology.

[9] Khashab MA, Yong E, Lennon AM (2011). EUS is still superior to multidetector computerized tomography for detection of pancreatic neuroendocrine tumors. Gastrointest Endosc.

[10] Klimistra DS, Modlin IR, Coppola D (2010). The pathologic classification of neuroendrocrine tumors a review of nomenclature, grading, and staging systems. Pancreas.

[11] Korse CM, Muller M, Taal BG (2011). Discontinuation of proton pump inhibitors during assessment of chromogranin A levels in patients with neuroendocrine tumours. Br J Cancer.

[12] Massironi S, Sciola V, Peracchi M, Ciafardini C, Spampatti MP, Conte D (2008). Neuroendocrine tumors of the gastro-entero-pancreatic system. World J Gastroenterol.

[13] National Comprehensive Cancer Network (2016). Guidelines Version Neuroendocrine Tumors of the Pancreas.

[14] Norton JA, Fraker DL, Alexander HR (2012). Value of surgery in patients with negative imaging and sporadic zollinger-ellison syndrome. Ann Surg.

[15] Peracchi M, Conte D, Gebbia C (2003). Plasma chromogranin A in patients with sporadic gastro-entero-pancreatic neuroendocrine tumors or multiple endocrine neoplasia type 1. Eur J Endocrinol.

[16] Rindi G, Klöppel G, Alhman H, Caplin M, Couvelard A, de Herder WW, Erikssson B, Falchetti A, Falconi M, Komminoth P, Körner M, Lopes JM, McNicol AM, Nilsson O, Perren A, Scarpa A, Scoazec JY, Wiedenmann B, Frascati Consensus Conference participantsEuropean Neuroendocrine Tumor Society (2006). TNM staging of foregut (neuro)endocrine tumors a consensus proposal including a grading system. Virchows Arch.

[17] Sorbye H, Strosberg J, Baudin E (2014). Gastroenteropancreatic high-grade neuroendocrine carcinoma. Cancer.

[18] Strosberg J, Gardner N, Kvols L (2009). Survival and prognostic factor analysis in patients with metastatic pancreatic endocrine carcinomas. Pancreas.

[19] Sundin A, Vullierme MP, Kaltsas G (2009). ENETS Consensus Guidelines for the Standards of Care in Neuroendocrine Tumors radiological examinations. Neuroendocrinology.

[20] Sundin A (2012). Radiological and nuclear medicine imaging of gastroenteropancreatic neuroendocrine tumours. Best Practice & Research. Clinical Obstetrics & Gynaecology.

[21] Surjan RC, Basseres T, Makdissi FF, Machado MAC, Ardengh JC (2017). Laparoscopic uncinatectomy: a more conservative approach to the uncinate process of the pancreas. Arq Bras Cir Dig.

[22] Yao JC, Hassan M, Phan A, Dagohoy C, Leary C, Mares JE, Abdalla EK, Fleming JB, Vauthey JN, Rashid A, Evans DB (2008). One hundred years after "carcinoid" epidemiology of and prognostic factors for neuroendocrine tumors in 35,825 cases in the United States. J ClinOncol.

